# A TRAb-First Diagnostic Strategy for Overt Hyperthyroidism: Diagnostic Performance and Implications for Reflex Testing

**DOI:** 10.3390/jcm15020445

**Published:** 2026-01-06

**Authors:** Petra Petranović Ovčariček, Alfredo Campennì, Federica D’Aurizio, Rosaria Maddalena Ruggeri, Luca Giovanella

**Affiliations:** 1 Department of Oncology and Nuclear Medicine, University Hospital Center Sestre Milosrdnice, 10000 Zagreb, Croatia; p.petranovic@gmail.com; 2 School of Medicine, University of Zagreb, 10000 Zagreb, Croatia; 3 Nuclear Medicine Unit, Biomedical, Dental Sciences and Morpho-Functional Imaging, University of Messina, 98100 Messina, Italy; alfredo.campenni@unime.it; 4 Laboratory for Clinical Pathology, Department of Laboratory Medicine, University Hospital of Udine, 33100 Udine, Italy; federica.daurizio@asufc.sanita.fvg.it; 5 Endocrinology Unit, Department of Human Pathology of Adulthood and Childhood DETEV, University of Messina, 98125 Messina, Italy; rosaria.ruggeri@unime.it; 6 Department of Nuclear Medicine and Thyroid Centre, Gruppo Ospedaliero Moncucco, Via Soldino 5, 6900 Lugano, Switzerland; 7 Clinic for Nuclear Medicine, University Hospital and University of Zürich, 8004 Zürich, Switzerland; 8 Clinical Chemistry and Endocrinology, Medysin, Sonic Suisse, 6004 Luzern, Switzerland

**Keywords:** Graves’ disease, TSH receptor antibodies, thyroid scintigraphy, reflex testing, hyperthyroidism diagnosis, cost-effectiveness, diagnostic strategy

## Abstract

**Background/Objectives**: To evaluate whether a TSH-receptor antibody (TRAb)-first, one-sample diagnostic strategy improves etiologic classification of overt hyperthyroidism compared with conventional pathways, and to assess its implications for imaging use, diagnostic accuracy, and cost efficiency. **Methods**: In this multicentre retrospective study, 274 adults with newly diagnosed overt hyperthyroidism underwent TRAb measurement, thyroid ultrasound, and scintigraphy during a single clinical encounter. Scintigraphy served as the functional reference standard. We compared the diagnostic performance of TRAb and ultrasound, modeled TRAb-first diagnostic algorithms, and estimated the potential impact of reflex TRAb testing on diagnostic workflow and resource use. **Results**: Graves’ disease (GD) accounted for 65% of cases. TRAb showed excellent diagnostic accuracy for GD (sensitivity 92.0%, specificity 96.0%; κ = 0.86) and markedly outperformed ultrasound (sensitivity 66.9%, specificity 62.5%; κ = 0.43). A TRAb-first pathway in which TRAb-positive patients are directly classified as GD and TRAb-negative patients undergo scintigraphy achieved 100% sensitivity, 95.8% specificity, and the lowest overall misclassification rate. Replacing scintigraphy with ultrasound in TRAb-negative patients substantially reduced specificity (~60%) and yielded significant overdiagnosis of GD. Ultrasound identified numerous nodules but detected only one low-risk carcinoma (malignancy rate: 1.2%), suggesting limited oncologic yield. A TRAb-first strategy would have avoided two-thirds of scintigraphies and minimized unnecessary imaging. **Conclusions**: A TRAb-first diagnostic approach offers the most accurate, efficient, and clinically appropriate pathway for etiologic assessment of overt hyperthyroidism. Scintigraphy should be reserved for TRAb-negative patients, while ultrasound should be used selectively for structural evaluation rather than as part of routine etiologic work-up. Reflex TRAb testing may further streamline care by enabling rapid, one-sample etiologic diagnosis and reducing resource use.

## 1. Introduction

Hyperthyroidism is a common endocrine disorder, affecting approximately 0.5–2% of adults in iodine-sufficient regions. Graves’ disease (GD) represents the most frequent etiology, followed by toxic multinodular goiter (MNTG) and toxic adenoma (TA) [1,2,3]. In a subset of patients, hyperthyroidism results from destructive thyroiditis or other less common conditions [4,5,6]. Accurate determination of the underlying etiology of hyperthyroidism is essential to enable timely and appropriate treatment [3,7,8]. While biochemical confirmation of overt hyperthyroidism relies on suppressed thyroid-stimulating hormone (TSH) with elevated thyroid hormones, the appropriate sequence of etiologic tests remains debated, and currently available international guidelines propose different approaches [9]. In particular, the American Thyroid Association (ATA) leaves local teams to perform thyroid scintigraphy and/or thyroid ultrasound and/or measure TSH-receptor antibody (TRAb), depending on local availability, expertise, and preferences [10]. The European Thyroid Association (ETA) advises TRAb measurement plus ultrasound in all patients, reserving scintigraphy for nodular disease [11] while the National Institute for Health and Care Excellence (NICE) recommends TRAb as the first-line etiologic test, followed by scintigraphy if the TRAb test is negative or inconclusive [12]. In many European healthcare systems, diagnostic pathways are further shaped by the availability of specialized endocrine or nuclear medicine thyroid clinics, where blood sampling, thyroid scintigraphy, and ultrasound are performed during a single visit (“one-stop-shop” model) [13]. While this organizational approach accelerates diagnosis, it frequently leads to routine multimodal testing, even when certain investigations may not be strictly indicated based on pre-test probability. In parallel, modern laboratory medicine increasingly adopts reflex testing strategies, whereby predefined biochemical patterns automatically trigger additional tests (e.g., TSH-reflex testing) [14]. Such approaches may simplify diagnostic workflows and reduce unnecessary follow-up visits and procedures [15,16]. Against this background, the present study aimed to evaluate whether a TRAb-first, single-sample diagnostic strategy improves etiologic classification of overt hyperthyroidism compared with conventional diagnostic pathways, and to assess its implications for imaging utilization, diagnostic accuracy, and cost efficiency.

## 2. Methods

### 2.1. Study Design and Population

This multicentre retrospective study was conducted at the thyroid clinics of the University Hospital of Messina (Italy) and Gruppo Ospedaliero Moncucco (Lugano, Switzerland), between 1 March 2024, and 30 August 2025. The study was performed in accordance with the Declaration of Helsinki on ethical principles for medical research involving human subjects [17]. Ethical approval was obtained from the Institutional Review Boards of Gruppo Ospedaliero Moncucco, Lugano (Switzerland) (ref. CE-GOM-24-00162) and the “G. Martino” University Hospital of Messina (Italy) (ref. 1917-bis-2022). Both centres operate as integrated thyroid clinics in which patients routinely undergo same-day blood sampling, thyroid scintigraphy, and ultrasonography as part of standard clinical care. No additional investigations were performed for research purposes; therefore, written informed consent was waived. All data were anonymized prior to analysis. Adult patients (≥18 years) with newly diagnosed overt primary hyperthyroidism—defined by suppressed TSH and elevated free thyroxine (fT4) and/or free triiodothyronine (fT3)—were eligible for inclusion. Exclusion criteria comprised pregnancy, prior thyroid disease, previous antithyroid treatment, and the use of medications or substances known to interfere with thyroid function.

### 2.2. Laboratory Assessment and Imaging Procedures

Thyroid function tests (TSH, fT4, fT3) and TRAb were measured in both centers using electrochemiluminescence immunoassay (ECLIA) on the Cobas^®^ platform according to the manufacturer’s instructions (Roche Diagnostics, Mannheim, Germany). Limit of Detection (LoD), Limit of Quantification (LoQ), measuring range and reference range were 0.005, 0.005, 0.005–100, 0.27–4.20 mUI/L for TSH; 0.6, 1.5, 0.6–50, 3.1–6.8 pmol/L for fT3; 0,5, 1.3, 0.5–100, 12.0–22.0 pmo/L for fT4; 0.8, 1.1, 0.8–40, ≤1.75 IU/L for TRAb, respectively [18,19,20]. Thyroid scintigraphy and thyroid ultrasound procedures were performed and evaluated by board-certified nuclear medicine physicians and/or endocrinologists according to current procedural guidelines [21,22], as previously described [23]. Non-autonomously functioning nodules (i.e., cold nodules) were assessed by ultrasound according to the European-Thyroid Imaging Reporting and Data System (EU-TIRADS), and a fine-needle aspiration cytology (FNAC) was performed in EU-TIRADS 5 nodules > 10 mm, EU-TIRADS 4 nodules > 15 mm, and EU-TIRADS 3 nodules > 20 mm, respectively [24]. Expert endocrine cytopathologists provided standardized cytological diagnoses classified according to the Bethesda Classification system [25].

### 2.3. Statistical Analysis

Because TSH, fT3, fT4, and TRAb values were not normally distributed, as assessed by the Kolmogorov–Smirnov test, continuous variables are presented as medians with interquartile ranges (IQRs). Thyroid scintigraphy was considered the functional reference standard for etiologic classification and was used to calculate sensitivity, specificity, positive predictive value (PPV), and negative predictive value (NPV) for TRAb and ultrasound. Agreement between diagnostic classifications was assessed using Cohen’s κ statistic. Group comparisons were performed using the Kruskal–Wallis test with Benjamini–Hochberg correction for multiple testing [26]. A two-sided *p* value < 0.05 was considered statistically significant. All statistical analyses were conducted using MedCalc Statistical Software (version 2025; MedCalc Software Ltd., Ostend, Belgium).

## 3. Results

A total of 274 patients with overt hyperthyroidism were included. The majority were female (209/274, 76.3%), with a median age of 46 years (IQR 43–54; mean 49.7 ± 14.5 years). Based on scintigraphy, the etiologic diagnosis was GD in 178 patients (65.0%), MNTG in 46 (16.8%), TA in 39 (14.2%), and destructive thyroiditis (DT) in 11 (4.0%). Patients with GD were significantly younger than those with MNTG or TA (both *p* < 0.001), whereas age did not differ significantly between GD and DT (Table 1).

### 3.1. TSH-Receptor Antibody

**Figure 1:**
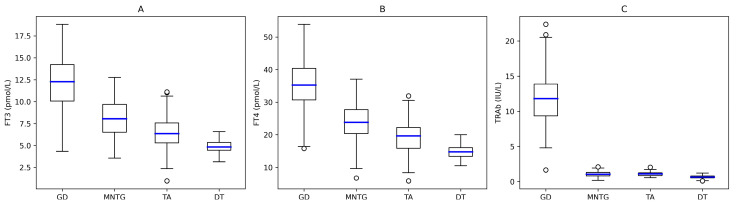
Distribution of free-thyroid hormones and TRAb across etiologies of overt hyperthyroidism. Boxplots display values on linear scale with median (blue line), IQR (empty squares), and whiskers (empty circles). (**A**) fT3 concentrations, (**B**) fT4 concentrations, (**C**) TRAb concentrations. **Legend.** GD, Graves’ Disease; MNTG, Toxic Multinodular Goiter; TA, Toxic Adenoma; DT, Destructive Thyroiditis.

Serum TSH levels were uniformly suppressed across all etiologic groups, making formal comparisons non-informative. Patients with Graves’ disease showed significantly higher fT3, fT4, and TRAb concentrations than those with other causes of thyrotoxicosis (all *p* < 0.001; Figure 1A–C). Agreement between TRAb and thyroid scintigraphy was near-perfect (Cohen’s κ = 0.86), whereas agreement between ultrasound and scintigraphy was only moderate (κ = 0.43). TRAb demonstrated excellent diagnostic performance for GD, with a sensitivity of 92.0% (95% CI 87.1–95.5), specificity of 96.0% (95% CI 89.8–98.9), PPV of 98.0% (95% CI 95.8–99.9), and NPV of 86.0% (95% CI 69.2–88.4). By contrast, ultrasound showed lower discriminative ability, with a sensitivity of 66.9% (95% CI 59.4–73.8), specificity of 62.5% (95% CI 51.7–72.5), PPV of 76.3% (95% CI 68.8–82.7), and NPV of 50.0% (95% CI 40.1–59.9). Overall, TRAb correctly classified 164 of 178 patients with GD and 92 of 96 non-GD patients, whereas ultrasound correctly classified 119 of 178 GD patients and 60 of 96 non-GD patients. Fourteen GD cases were TRAb false-negative and four non-GD cases—each with nodular autonomy—were false-positive. All discordant results clustered near the diagnostic cut-off (1.75 IU/L), with mildly elevated titres in false positives (median 2.1 IU/L) and borderline sub-threshold values in false negatives (median 1.5 IU/L) (Figure 1C).

### 3.2. Thyroid Ultrasound

Thyroid ultrasonography identified 214 nodules in 104 of 274 patients with hyperthyroidism (38%). Of these, 135 nodules were scintigraphically hyperfunctioning, while 79 were cold nodules detected in patients with GD (n = 22) and MNTG (n = 57). Six patients underwent fine-needle aspiration cytology (FNAC) based on suspicious ultrasound features combined with nodule size criteria. As summarized in Table 2, cytology was benign in three cases (2 GD, 1 MNTG), whereas three patients proceeded to surgery due to highly suspicious (n = 1) or indeterminate (n = 2) cytology. One papillary thyroid carcinoma (PTC) was identified in a patient with MNTG; the remaining surgical specimens were benign (1 GD, 1 MNTG). The detected PTC measured 12 mm in maximum diameter and showed no aggressive features or lymph node involvement (pT1bN0, low-risk ATA classification). Overall, the malignancy rate among cold nodules was 1.2%.

### 3.3. Comparison of Diagnostic Strategies

Finally, two TRAb-first diagnostic pathways for etiologic assessment of overt hyperthyroidism were compared. In both strategies, TRAb-positive patients were classified as having Graves’ disease, while TRAb-negative patients underwent either thyroid scintigraphy or ultrasonography. When scintigraphy was used as the second-line test, the TRAb–scintigraphy pathway achieved 100% sensitivity (178/178 GD correctly identified) and 95.8% specificity (92/96 non-GD correctly classified), with a PPV of 97.8% and an NPV of 100%. Only four patients with nodular autonomy were misclassified, and no cases of GD were missed. In contrast, substituting scintigraphy with ultrasound in TRAb-negative patients substantially reduced diagnostic performance. Although sensitivity remained high (97.4%), specificity declined markedly to 60.0%, resulting in a high false-positive rate (approximately 38 of 96 non-GD patients misclassified as GD). Predictive values were also inferior (PPV ≈ 81.9%, NPV ≈ 92.4%), and overall misclassification increased from 1.5% with scintigraphy to approximately 15.7% with ultrasound. These findings indicate that, within a TRAb-first diagnostic framework, scintigraphy is the optimal second-line investigation for TRAb-negative hyperthyroidism, whereas ultrasound leads to substantial overdiagnosis of GD and reduced diagnostic accuracy.

## 4. Discussion

**Figure 2:**
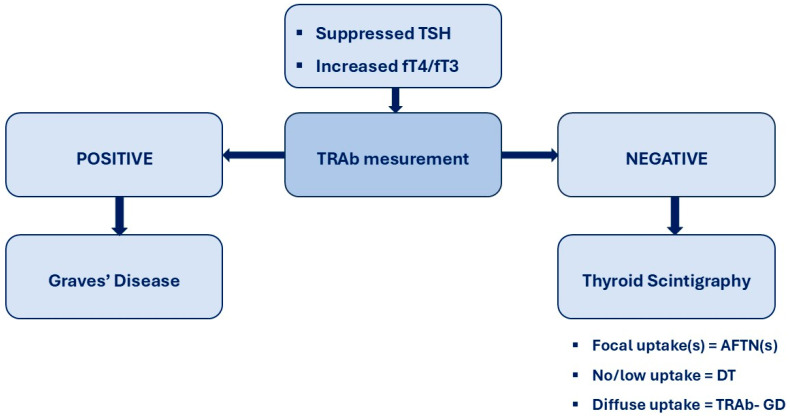
TRAb-first diagnostic flowchart for hyperthyroidism. In patients presenting with biochemical overt hyperthyroidism, serum TSH-receptor antibodies (TRAb) are measured as the initial diagnostic step. A positive TRAb result establishes the diagnosis of Graves’ disease and allows prompt initiation of disease-specific management without further etiological testing. A negative or equivocal TRAb result prompts second-line evaluation with thyroid scintigraphy to autonomously functioning thyroid nodule(s) [AFTN(s)] or destructive thyroiditis [DT].

Timely and accurate etiologic differentiation of overt hyperthyroidism is essential for optimal clinical management, as emphasized by both European and American Thyroid Association guidelines [10,11]. In this multicentre cohort evaluated within a standardized “one-stop-shop” diagnostic framework, TRAb showed the highest diagnostic accuracy for distinguishing Graves’ disease (GD) from non-GD etiologies, outperforming ultrasonography and closely approximating thyroid scintigraphy, which is the functional reference standard. In accordance with guideline recommendations, scintigraphy was selected as the reference method because it directly assesses thyroid functional activity and reliably differentiates autoimmune hyperthyroidism from thyroid autonomy at initial presentation. Although scintigraphy may underestimate very early autoimmune disease or incompletely characterize mixed functional patterns, alternative composite reference standards based on clinical evolution or treatment response are retrospective, less standardized, and prone to incorporation bias. Within this pragmatic framework, TRAb demonstrated excellent sensitivity and specificity and near-perfect agreement with scintigraphy, supporting its role as a first-line etiologic test in overt hyperthyroidism. By contrast, ultrasonography showed only moderate agreement with scintigraphy and substantially lower discriminative performance. As a primarily morphological technique, ultrasound identifies structural rather than functional abnormalities, which likely explains the frequent overdiagnosis of GD in patients with nodular autonomy, where heterogeneous echogenicity and increased vascularity may mimic autoimmune patterns. Although ultrasonography detected numerous nodules, its clinical yield for malignancy was very low, with only one low-risk papillary thyroid carcinoma identified among 79 cold nodules (1.2%). Importantly, all examinations were performed by experienced specialists using standardized criteria, suggesting that misclassification was not primarily driven by operator dependency and may be even greater in less specialized settings. Collectively, these findings do not support routine ultrasound in all TRAb-negative patients; a targeted approach limited to selected clinical indications appears more appropriate [27]. Overall, our data indicate that a TRAb-alone, frontline diagnostic strategy is both feasible and highly accurate for discriminating GD from other causes of overt hyperthyroidism, showing near-perfect agreement with scintigraphy and clearly outperforming ultrasonography. These results align with recent studies demonstrating that contemporary TRAb immunoassays provide excellent diagnostic performance and reliably support an antibody-first strategy, with scintigraphy reserved for TRAb-negative cases or suspected thyroid autonomy [9,20,28], as summarized in Figure 2.

Reports describing limitations of TRAb-based diagnosis largely concern uncommon scenarios, including very early or mild autoimmune hyperthyroidism, seronegative or borderline-antibody GD, or mixed functional patterns. Nishihara et al. identified TRAb-negative GD or TSH receptor mutations in a small subset of hyperthyroid patients evaluated over a 10-year period [29], while Lupo et al. reported lower TRAb positivity in heterogeneous cohorts that included treated patients and those with subclinical disease [30]. In contrast, our study focused exclusively on patients with newly diagnosed overt hyperthyroidism evaluated at first presentation, involving a clinically homogeneous setting in which TRAb demonstrated substantially higher sensitivity and near-perfect agreement with scintigraphy. Importantly, in cases of GD with negative TRAb results, thyroid scintigraphy remains an essential second-line investigation, typically demonstrating diffuse hyperfunction and excluding thyroid autonomy or destructive thyroiditis, thereby guiding appropriate management [31,32,33]. Within this sequential framework, TRAb functions effectively as a first-line test while etiologic assessment remains embedded in comprehensive clinical judgment. The TRAb-first strategy may be further optimized through reflex testing, whereby TRAb measurement is automatically added when overt biochemical hyperthyroidism is detected. This one-sample diagnostic pathway may reduce additional visits, phlebotomies, and unnecessary imaging, aligning with modern laboratory medicine practices. Nevertheless, analytical variability among TRAb assays, healthcare-system constraints, and the clustering of false-positive and false-negative results near the diagnostic cut-off suggest that strict binary interpretation may be suboptimal. Introduction of an assay-specific indeterminate (“gray-zone”) range triggering second-line scintigraphy may better reflect biological and analytical variability and improve diagnostic accuracy. Finally, the retrospective design and the specialized “one-stop-shop” setting may limit generalizability. Prospective studies in broader clinical environments, including formal health economic evaluation, are required to assess the real-world applicability, scalability, and cost-effectiveness of a TRAb-first diagnostic strategy.

## 5. Conclusions

TRAb demonstrated superior diagnostic accuracy for etiologic classification of overt hyperthyroidism, supporting a TRAb-first, and potentially reflex, diagnostic strategy, with thyroid scintigraphy reserved for TRAb-negative cases or suspected thyroid autonomy. Prospective studies and formal health economic evaluations are warranted to validate its clinical effectiveness and cost efficiency.

## Figures and Tables

**Table 1:** Baseline demographic and clinical characteristics of patients with overt hyperthyroidism, stratified by scintigraphy-based etiology.

Diagnosis	n (%)	Female, n (%)	Male, n (%)	Age, Median (IQR)
GD	178 (65.0)	143 (80.3)	35 (19.7)	44 (35–54) *
MNTG	46 (16.8)	31 (67.4)	15 (32.6)	64 (58–72) **
TA	39 (14.2)	25 (64.1)	14 (35.9)	54 (47–63) **^#^**
DT	11 (4.0)	10 (90.9)	1 (9.1)	43 (38–50) **^##^**
All	274 (100)	209 (76.3)	65 (23.7)	46 (43–54)

**Legend.** GD, Graves’ Disease; MNTG, Toxic Multinodular Goiter; TA, Toxic Adenoma; DT, Destructive Thyroiditis, n, number; IQR, interquartile range. Age: GD * vs. MNTG **, *p* < 0.001; GD * vs. TA ^#^, *p* < 0.001; GD * vs. ^##^ DT, *p* = ns; other comparisons, *p* = ns.

**Table 2:** Ultrasonographic, cytological, and histopathological characteristics of suspicious cold thyroid nodules.

Diagnosis	Sex	Age	Size	EU-TIRADS	Bethesda	Pathology
Graves’ Disease	F	27	21	EU-TIRADS 3	II	No surgery
Graves’ Disease	F	33	16	EU-TIRADS 4	II	No surgery
Graves’ Disease	F	56	12	EU-TIRADS 4	III	Hyperplastic nodule
MNTG	M	67	27	EU-TIRADS 3	IV	Follicular adenoma
MNTG	F	59	12	EU-TIRADS 5	V	PTC
MNTG	F	74	24	EU-TIRADS 3	II	No surgery

**Legenda.** MNTG, multinodular toxic goiter; F, female; M, male; EU-TIRADS, European-Thyroid Imaging Reporting and Data System; PTC, papillary thyroid carcinoma. Age is expressed in years, size in millimeters (mm).

## Data Availability

Data are available on reasonable request to the corresponding author.
